# Functional Materials from Nanocellulose: Utilizing Structure–Property Relationships in Bottom‐Up Fabrication

**DOI:** 10.1002/adma.202000657

**Published:** 2020-04-08

**Authors:** Kevin De France, Zhihui Zeng, Tingting Wu, Gustav Nyström

**Affiliations:** ^1^ Laboratory for Cellulose and Wood Materials Swiss Federal Laboratories for Materials Science and Technology (Empa) Überlandstrasse 129 Dübendorf 8600 Switzerland; ^2^ Department of Health Science and Technology ETH Zürich Schmelzbergstrasse 9 Zürich 8092 Switzerland

**Keywords:** biopolymer aerogels, cellulose nanocrystals, cellulose nanofibrils, self‐assembly, structured biomaterials

## Abstract

It is inherently challenging to recapitulate the precise hierarchical architectures found throughout nature (such as in wood, antler, bone, and silk) using synthetic bottom‐up fabrication strategies. However, as a renewable and naturally sourced nanoscale building block, nanocellulose—both cellulose nanocrystals and cellulose nanofibrils—has gained significant research interest within this area. Altogether, the intrinsic shape anisotropy, surface charge/chemistry, and mechanical/rheological properties are some of the critical material properties leading to advanced structure‐based functionality within nanocellulose‐based bottom‐up fabricated materials. Herein, the organization of nanocellulose into biomimetic‐aligned, porous, and fibrous materials through a variety of fabrication techniques is presented. Moreover, sophisticated material structuring arising from both the alignment of nanocellulose and via specific process‐induced methods is covered. In particular, design rules based on the underlying fundamental properties of nanocellulose are established and discussed as related to their influence on material assembly and resulting structure/function. Finally, key advancements and critical challenges within the field are highlighted, paving the way for the fabrication of truly advanced materials from nanocellulose.

## Graphical abstract

As a natural and renewable building block for bottom‐up fabrication, nanocellulose has gained considerable interest for the assembly of sophisticated materials with advanced functionality. The organization of nanocellulose into aligned, porous, and fibrous materials is highlighted, focusing on the inherent material properties enabling the formation of such structures, along with discussing key advancements and critical challenges within the field.

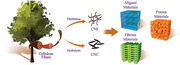

## 1 Introduction

**Figure 1:**
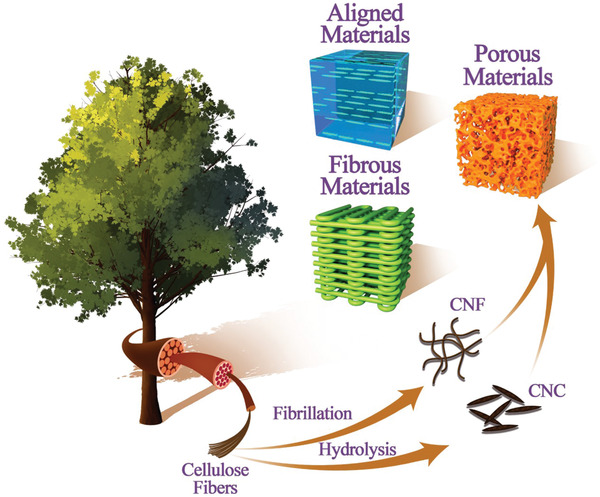
Schematic illustration of the production of nanocellulose (CNC and CNF) from natural wood sources, and their hierarchical organization into new aligned, porous, and fibrous materials through bottom‐up fabrication.

Nature has the innate ability to assemble sophisticated hierarchical structures on multiple length scales with remarkable functionalities from simple nanoscale building blocks such as proteins and carbohydrates. This relationship between a material's macroscale functionality and its nanoscale building blocks is readily evidenced throughout nature, such as in structures including cellulose (wood), chitin (crustacean shells), and silk (spider webs).^[^ ^1^ ^]^ Bottom‐up fabrication acts to mimic this hierarchical assembly found commonly in nature in order to develop advanced/next‐gen materials with unique properties and functionalities.^[^ ^1^, ^2^ ^]^ In keeping with the idea of mimicking nature, the use of renewable/naturally sourced building blocks for the development of hierarchical structures has recently been a central theme within the realm of bottom‐up fabrication. Nanocellulose, comprising both cellulose nanocrystals (CNCs) and cellulose nanofibrils (CNFs) (**Figure** **1**), is one such building block, which is made up of cellulose, the most abundant renewable polymer on earth.

CNCs and CNFs have garnered significant research interest in recent years, with widespread target applications across several industries including the biomedical, energy storage, packaging, composite materials, and specialty chemicals sectors.^[^ ^3^, ^4^, ^5^ ^]^ These highly crystalline, high aspect ratio nanoparticles are composed of linear homopolymers of β (1–4) linked D‐glucose units and exhibit impressive mechanical properties and tunable surface chemistries. Given the high strength, dimensional anisotropy, and natural sourcing of both CNCs and CNFs, the use of nanocellulose as a functional building block for developing hierarchical assemblies has attracted significant research interest. Due to this widespread interest in nanocellulose, several reviews have previously been published covering the material properties, production, processing, characterization strategies, chemical modifications, and potential applications of CNCs and CNFs, to which we direct any interested reader for additional information.^[^ ^2^, ^3^, ^4^, ^5^, ^6^, ^7^, ^8^, ^9^, ^10^, ^11^, ^12^, ^13^, ^14^, ^15^, ^16^, ^17^, ^18^, ^19^ ^]^

CNCs are rigid rod‐shaped particles with average length and cross‐section of 100–200 and 5–20 nm, respectively, and are generally extracted from sources such as wood pulp or cotton through an acid hydrolysis process.^[^ ^20^, ^21^ ^]^ Although several different acids and oxidizing agents have been used to produce CNCs with varying surface chemistries (e.g., carboxyl groups,^[^ ^22^, ^23^, ^24^, ^25^ ^]^ phosphate half‐esters,^[^ ^26^ ^]^ or uncharged^[^ ^27^ ^]^), commercial production has generally focused on sulfuric acid hydrolysis, leaving negatively charged sulfate half‐ester groups on the surface.^[^ ^28^ ^]^ Conversely, CNFs are relatively more flexible/kinked^[^ ^29^ ^]^ and longer than their CNC counterparts (average length > 1 μm), and are produced through high energy mechanical homogenization of wood pulp. This fibrillation process is often combined with enzymatic treatments,^[^ ^30^ ^]^ 2,2,6,6‐tetramethylpiperidine‐1‐oxyl (TEMPO) oxidation,^[^ ^25^ ^]^ or other chemical modifications (e.g., carboxymethylation^[^ ^31^ ^]^), and as such, commercially produced CNFs can possess a wide variety of both surface chemistries and charge densities. Notably, without chemical/enzymatic treatments, the fibrillation process can be extremely energy intensive, and can lead to CNF with low surface charge and a higher residual hemicellulose/lignin content.^[^ ^32^ ^]^ As such, these long fibrils have a lower degree of crystallinity than CNCs (70% vs > 85%),^[^ ^33^ ^]^ and form physically entangled networks/gels at much lower concentrations (≈1 wt% vs ≈10 wt%).

Herein, this report highlights the current status, future outlook, and ongoing challenges associated with the bottom‐up fabrication of functional materials (including hydrogels, aerogels, films, fibers, and other composite materials) containing nanocellulose. In particular, we emphasize the critical roles that nanocellulose itself plays (originating from unique characteristics including shape anisotropy, aspect ratio, surface area, diverse surface chemistry, and excellent mechanical properties) in the structure–property relationships for each type of assembly, including porosity, surface area, aspect ratio, nanoparticle alignment, charge, aggregation, etc. Functional nanocellulose materials are categorized by the primary type of structuring employed during preparation, namely, nanocellulose alignment‐induced structuring (Section 2), and material processing‐induced structuring (Section 3).

## 2 Nanocellulose Alignment‐Induced Structuring

The functionality of many high‐performance biomaterials is a direct result of the alignment of its components; therefore controlling nanoparticle orientation is of high importance when developing biomimetic materials. For example, material properties such as mechanics, optics, and swelling may be enhanced upon successful nanoparticle alignment. Due to the anisotropic rod shape of both CNCs and CNFs, it is relatively easy to orient them using a variety of external forces.^[^ ^34^ ^]^ CNCs and CNFs can be actively aligned in a uniaxial manner using shear forces via a variety of different techniques including spin coating,^[^ ^35^ ^]^ dip coating,^[^ ^36^, ^37^ ^]^ extrusion/spinning,^[^ ^38^, ^39^, ^40^ ^]^ stretching/drawing,^[^ ^41^, ^42^ ^]^ and casting.^[^ ^43^, ^44^, ^45^ ^]^ Furthermore, due to their enhanced crystallinity, diamagnetic susceptibility, and structural chirality, CNCs can co‐operatively assemble into chiral nematic structures.^[^ ^46^, ^47^ ^]^ This section highlights the various techniques used to control nanocellulose alignment and prepare structured materials with a high degree of nanoparticle order.

### 2.1 Uniaxial/Nematic Alignment

The shear alignment of CNCs was first investigated by Orts et al., who demonstrated that as the shear rate was increased, overall CNC orientation correspondingly increased.^[^ ^48^ ^]^ This process of shearing suspensions of nanocellulose to align the individual fibers has been well‐studied over the past few decades.^[^ ^49^ ^]^ Recent work in the Davis lab has demonstrated the preparation of microelectromechanical systems (MEMS) from shear‐casted aligned CNC films.^[^ ^50^ ^]^ The resulting MEMS demonstrated both anisotropic mechanical and optical properties, which were tuned by controlling the direction of CNC alignment with respect to the long axis of the MEMS device.^[^ ^50^ ^]^ Similarly, the MacLachlan lab has demonstrated the preparation of chemically crosslinked composites with uniaxially aligned CNCs via shearing of the precursor suspension between glass slides and subsequent photo‐ or thermal‐initiated polymerization.^[^ ^51^, ^52^ ^]^ These sheared composites show ionic strength‐dependent anisotropic swelling profiles, whereby an increase in ionic strength leads to significantly higher shrinking in the direction perpendicular to CNC alignment, and a corresponding increase in the overall birefringence of the hydrogel; a phenomenon which could be useful in sensing applications.^[^ ^51^ ^]^ Work in the Walther lab has demonstrated the shear alignment of composite films of carboxymethyl cellulose and CNCs prepared *after* solution casting.^[^ ^53^ ^]^ In this work, composite films containing randomly oriented CNCs were placed in a uniaxial drawing device in order to controllably stretch the films; increasing the draw ratio leads to increased CNC orientation (determined by electron microscopy and wide angle X‐ray diffraction (XRD)), and in turn increased Young's modulus, yield stress, and tensile strength of the composite films.^[^ ^53^ ^]^ Spin coating is another simple method of shear alignment that has been used effectively to create *radially* aligned nanocellulose films; whereby, the higher the speed of spin coating, the higher degree of alignment achievable. In a series of work, the Eichhorn lab has shown that spin‐coated films of radially oriented CNCs are capable of directing the growth and differentiation of C2C12 mouse myoblast cells, with a higher degree of CNC alignment leading to increased myoblast orientation.^[^ ^54^, ^55^ ^]^

**Figure 2:**
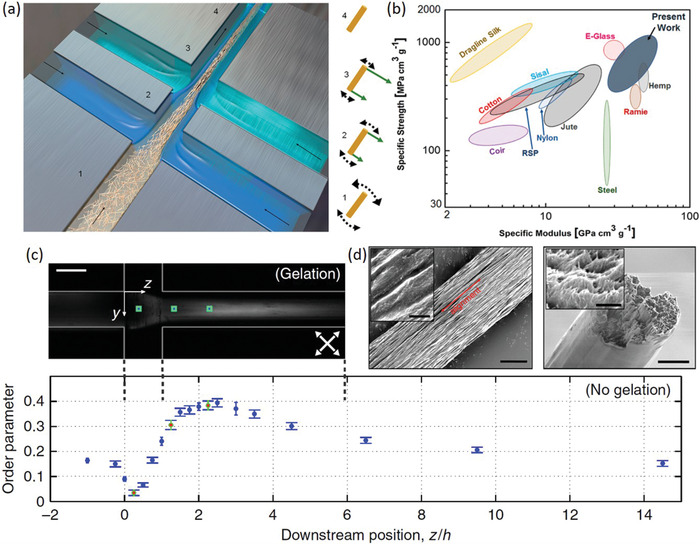
a) Schematic of the flow focusing technique to produce filaments with oriented CNFs. CNFs at position 1 are poorly oriented due to electrostatic repulsion and Brownian motion; CNFs at position 2 and 3 are subject to increased hydrodynamic‐induced alignment due to increased flow; and CNFs at position 4 are arrested due to increased ionic strength and overall gelation. b) The highly oriented filaments prepared in this work represent some of the highest strength and stiffness values achievable for any natural and commercial bio‐based materials. c) Image of the microfluidic flow focusing channel used to produce aligned filaments. d) Scanning electron microscopy (SEM) images of the produced filaments, and e) CNF orientational order parameter as a function of downstream position within the flow focusing channel. a,b,d) Adapted with permission.^[^ ^64^ ^]^ Copyright 2018, American Chemical Society. c,e) Adapted with permission.^[^ ^63^ ^]^ Copyright 2014, Springer Nature.

The alignment of nanocellulose has also been demonstrated using a variety of different spinning techniques.^[^ ^39^ ^]^ Chen et al. demonstrated dry spun cellulose acetate (CA)–CNC composite fibers, whereby the shear rates generated during the spinning process were sufficient to uniaxially align the CNCs, as characterized via XRD.^[^ ^56^ ^]^ They further demonstrated that increasing the CNC loading within the fiber composites from 1 wt% up to 34 wt% resulted in an increase in overall CNC orientation (order parameter ≈0.05–0.65) and simultaneous increase in elastic modulus (2.5–14.0 GPa) and tensile strength (67.7–160.3 MPa); however it is challenging to decouple the effects of increased CNC concentration and increased orientation on the resulting mechanical properties.^[^ ^56^ ^]^ Endo et al. prepared wet‐spun poly(vinyl alcohol) (PVA)–CNF composite fibers, demonstrating that increasing the fiber draw ratio from 3 to 20 resulted in a corresponding increase in fiber tensile modulus (5–57 GPa), attributed to increased orientation of both CNF and PVA chains.^[^ ^57^ ^]^ Similar relationships between draw ratio (spinning rate), CNF alignment, and mechanical strength have also been reported for pure wet‐spun CNF fibers,^[^ ^58^, ^59^ ^]^ other CNF‐composite fibers,^[^ ^60^ ^]^ and regenerated cellulose–CNF films.^[^ ^61^ ^]^ Interestingly, Lundahl et al. used a coaxial wet‐spinning approach to prepare composite filaments; by varying both the type of polymer in the shell and the solvent in the coagulation bath, the degree of CNF orientation could be controlled.^[^ ^62^ ^]^ The authors attributed increased CNF alignment to increase affinity of the core/shell materials and to increase rate of coagulation, respectively. Finally, the Söderberg lab has utilized a flow‐focusing technique, coupled with ion‐mediated gelation^[^ ^63^ ^]^ and optional 1,2,3,4‐butanetetracarboxylic acid (BTCA) chemical crosslinking^[^ ^64^ ^]^ to simultaneously orient and gel CNF suspensions into organized filaments (**Figure** **2**). In the first case, both gelation rate (controlled by ionic strength) and flow rate had significant impacts on CNF alignment (order parameter of 0.5 vs 0.38 when ionic strength halved, and vs 0.39 when flow rate decreased by 40%) and resulting filament modulus (18.0 GPa vs 12.4 and vs 12.8 GPa for respective conditions).^[^ ^63^ ^]^ While in the second case, it was demonstrated that CNFs of shorter lengths were able to orient (and disorient, in the absence of appropriate crosslinking) faster and to a greater degree due to their increased rotary and Brownian motion, respectively, versus CNFs of longer lengths. Here, increased alignment again led to increases in the mechanical properties of the produced filaments.^[^ ^64^ ^]^

More recently, extrusion‐based techniques such as 3D printing have demonstrated the potential to align nanocellulose during the printing process, and as such have gained research interest for the preparation of composite materials with complex architectures.^[^ ^65^ ^]^ Recent work by Hausmann et al. investigated the dynamics of CNC alignment via direct ink writing to create anisotropic 3D‐printed objects.^[^ ^66^ ^]^ It was determined that both the inherent rheology/viscosity of the ink as well as the flow architecture during printing have significant effects on CNC alignment; in general, alignment time was found to be linearly dependent on the applied shear rate, and nozzle geometries designed to produce both shear and extensional forces led to increased orientation overall.^[^ ^66^ ^]^ Utilizing this knowledge, the authors were able to prepare both pure CNCs and CNC‐composite materials with a high degree of alignment along the printing direction, and corresponding anisotropic mechanical properties.^[^ ^67^ ^]^ Prince et al. demonstrated the preparation of anisotropic chemically crosslinked CNC‐gelatin hydrogel sheets with compositional gradients using a microfluidic sheet extruding device.^[^ ^68^ ^]^ By varying both the ratio of CNC:gelatin and their overall concentrations input into the microfluidic device, compositional gradients could be created both parallel and perpendicular to the direction of extrusion (and thus CNCs orientation).^[^ ^68^ ^]^

Due to their anisotropic diamagnetic susceptibility (arising from the summation of magnetic dipoles of individual bonds within oriented cellulose polymer chains in a CNC), CNCs can be aligned in the presence of external electromagnetic fields.^[^ ^69^ ^]^ Although electric field alignment generally leads to highly oriented CNCs (or CNFs^[^ ^70^ ^]^) in the direction of the applied field, CNCs should be suspended in nonpolar solvents to avoid complications surrounding the high conductivity of water.^[^ ^71^, ^72^, ^73^, ^74^ ^]^ For magnetic fields however, CNC orientation/ordering depends on the concentration of suspension; for dilute systems, CNCs individually orient in a linear (nematic) fashion with their long axis perpendicular to the applied magnetic field,^[^ ^69^ ^]^ for semi‐dilute systems with increased CNC particle–particle interactions, CNCs tend to collectively orient in a nematic fashion in order to minimize translational entropy,^[^ ^75^ ^]^ while for concentrated systems, CNCs spontaneously co‐operatively self‐assemble into liquid crystalline (chiral nematic) tactoids with the helical director of the tactoid‐oriented parallel to the applied field (discussed in detail in Section 2.2).^[^ ^48^, ^76^ ^]^ For both cases, higher field strength and higher CNC aspect ratio have been demonstrated to lead to higher overall orientation.^[^ ^73^, ^77^ ^]^

**Figure 3:**
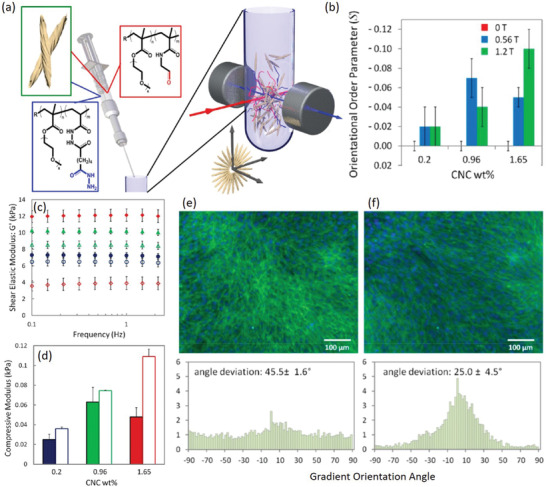
a) Schematic of injectable hydrogels prepared from POEGMA and in situ magnetically aligned CNCs. b) The degree of CNC orientation (*S*) as determined via SAXS is dependent on both magnetic field strength and CNC wt%. c) Hydrogels with aligned CNCs (unfilled symbols) show a decreased shear modulus and d) increased compressive modulus versus hydrogels with unaligned CNCs (filled symbols) for 0.2 (blue), 0.96 (green), and 1.65 (red) wt% CNC. e) Differentiated myotubes on hydrogels with unaligned, and f) magnetically aligned CNCs; image analysis on the myotube F‐actin filaments demonstrates their preferential orientational order when cultured on hydrogels with aligned CNCs versus hydrogels with unaligned CNCs. a–f) Adapted with permission.^[^ ^80^ ^]^ Copyright 2017, American Chemical Society.

Kvien and Oksman demonstrated that nematic CNC alignment is still possible within composite materials, whereby cast films from magnetically aligned CNCs and PVA demonstrated anisotropic mechanical performance, with a storage modulus of 6.2 GPa in the direction of alignment and 4.2 GPa in the perpendicular direction.^[^ ^78^ ^]^ Pullawan et al. demonstrated similar trends in mechanical reinforcement for CNC‐regenerated cellulose composite films.^[^ ^79^ ^]^ Finally, in a series of work, De France et al. prepared injectable nanocomposite hydrogels from chemically crosslinkable poly(oligo ethyleneglycol methacrylate) (POEGMA) and physically incorporated CNCs, whereby CNC alignment was possible in situ using a weak magnetic field (up to 1.2 T; achievable with a rare earth magnet) (**Figure** **3**).^[^ ^80^, ^81^, ^82^ ^]^ CNC alignment was quantified using small‐angle X‐ray scattering (SAXS), where the degree of orientation could be increased by increasing both the magnetic field strength (0–1.2 T) and the CNC concentration within the hydrogel network (0.2–1.65 wt%).^[^ ^80^ ^]^ While only moderate CNC alignment was possible due to both the viscosity of the hydrogel precursor suspension restricting CNC mobility, and the relatively quick in situ gelation (<10 min) minimizing the time available for CNC reorientation, significant differences in the physical properties of hydrogels with aligned and unaligned CNCs were demonstrated.^[^ ^80^ ^]^ For 1.65 wt% CNC hydrogels, the compressive modulus observed in the direction perpendicular to CNC alignment was increased (0.11 kPa vs 0.05 kPa) while the shear modulus observed parallel to CNC alignment was decreased (4 kPa vs 12 kPa) relative to hydrogels with unaligned CNCs.^[^ ^80^ ^]^ Finally, directional growth and differentiation of C2C12 mouse myoblast cells was demonstrated for hydrogels with aligned CNCs, which was not possible for hydrogels with unaligned CNCs.^[^ ^80^ ^]^

### 2.2 Chiral Nematic Order

Above a critical concentration (and below the onset of physical gel formation), CNCs will spontaneously phase separate/self‐assemble into a biphasic regime in order to maximize the trade‐off between reduced rotational and increased translational entropies of individual CNCs, as described by Onsager et al.^[^ ^46^, ^83^, ^84^ ^]^ This biphasic regime constitutes an upper isotropic phase and a lower anisotropic/chiral nematic phase.^[^ ^85^, ^86^, ^87^ ^]^ Properties such as aspect ratio, ionic strength, surface charge, and polymer grafting have all been shown to affect the critical concentration for self‐assembly and the overall CNC phase behavior.^[^ ^46^, ^88^, ^89^ ^]^ As many high‐performance natural biocomposites arise from chiral nematic self‐assembly,^[^ ^46^ ^]^ controlling CNC phase behavior and manipulating/preserving the inherent chiral nematic order are of high interest for developing next‐generation biomimetic materials.^[^ ^90^ ^]^

**Figure 4:**
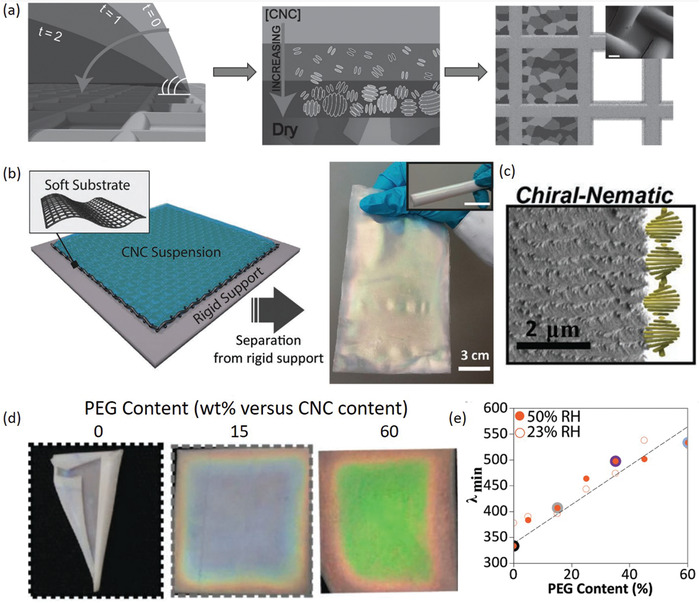
a) Schematic of the EISA process to prepare CNC films on porous mesh substrates. As the water evaporates, the CNC concentration increases promoting their self‐assembly into chiral nematic tactoids, eventually merging to form a bulk chiral nematic phase. b) The resulting films are highly elastic, and exhibit the characteristic iridescence and c) chiral nematic structuring, as evidenced by SEM. d) The incorporation of PEG as a plasticizer leads to increased film uniformity and e) an increase in the peak reflectance wavelength. a) Adapted with permission.^[^ ^103^ ^]^ Copyright 2017, Wiley‐VCH. b–d) Adapted with permission.^[^ ^104^ ^]^ Copyright 2019, Wiley‐VCH.

In 1992, Revol et al. first described the preservation of chiral nematic order following evaporation to form CNC films with structural color;^[^ ^86^ ^]^ a process that has since been termed “evaporation‐induced self‐assembly” (EISA). The structural color within these films arises due to both the CNC interparticle spacing and the twist/angular offset between each CNC layer, and can be controlled by varying the ionic strength, CNC surface charge, or by applying external electromagnetic fields, providing a versatile handle for controlling material properties.^[^ ^91^, ^92^ ^]^ Since Revol's seminal work, several groups have demonstrated that changing parameters relating to the EISA process (e.g., rate/geometry of evaporation, substrate, ionic strength)^[^ ^93^, ^94^, ^95^ ^]^ and inherent to the CNCs themselves (e.g., pre‐treatment conditions, surface chemistry, aspect ratio)^[^ ^96^ ^]^ can have significant effects on the resulting film physical properties and chiral nematic order. For example, Chen et al. demonstrated that by increasing the rate of evaporation via vacuum filtration, the birefringence of the resulting CNC films could be controlled.^[^ ^97^ ^]^ Natarajan et al. investigated the effects of CNC aspect ratio by using mixtures of CNCs with short (derived from wood pulp) and long (derived from tunicates) aspect ratios to prepare chiral nematic films.^[^ ^98^ ^]^ In general, by increasing the mass fraction of long aspect ratio CNCs within the films, both modulus and tensile strength were greatly enhanced while the peak reflectance wavelength was decreased due to a decrease in the chiral nematic pitch.^[^ ^98^ ^]^ Notably, CNC films prepared through EISA are often extremely brittle, and therefore the incorporation of polymers/plasticizers has been employed to increase film elasticity (and provide other material properties based on the composite material chosen) without destroying the chiral nematic organization.^[^ ^99^, ^100^, ^101^, ^102^ ^]^ In a series of work, Tardy et al. demonstrated the self‐assembly and microtemplating of CNC films by using porous meshes rather than smooth substrates (**Figure** **4**).^[^ ^103^, ^104^ ^]^ Here, the use of polyethylene glycol (PEG) as a plasticizer helped to reduce the build‐up of residual stresses during EISA, resulting in increased film uniformity and reduced buckling.^[^ ^104^ ^]^ Importantly, these films retained the elasticity of the porous mesh substrates while still exhibiting the birefringence and iridescence characteristic of CNC films formed via EISA.^[^ ^103^, ^104^ ^]^

Several groups have demonstrated the use of EISA‐based films for humidity sensing, whereby an increase in humidity leads to increased water sorption within the CNC film, and an increase in the chiral nematic pitch, resulting in a distinct yet reversible red‐shift of the film's structural color.^[^ ^105^, ^106^ ^]^ Interestingly, Zhao et al. demonstrated this phenomenon within printed microfilm arrays, proposing the use of such responsive films for decorative, sensing, or anticounterfeiting applications.^[^ ^107^ ^]^

**Figure 5:**
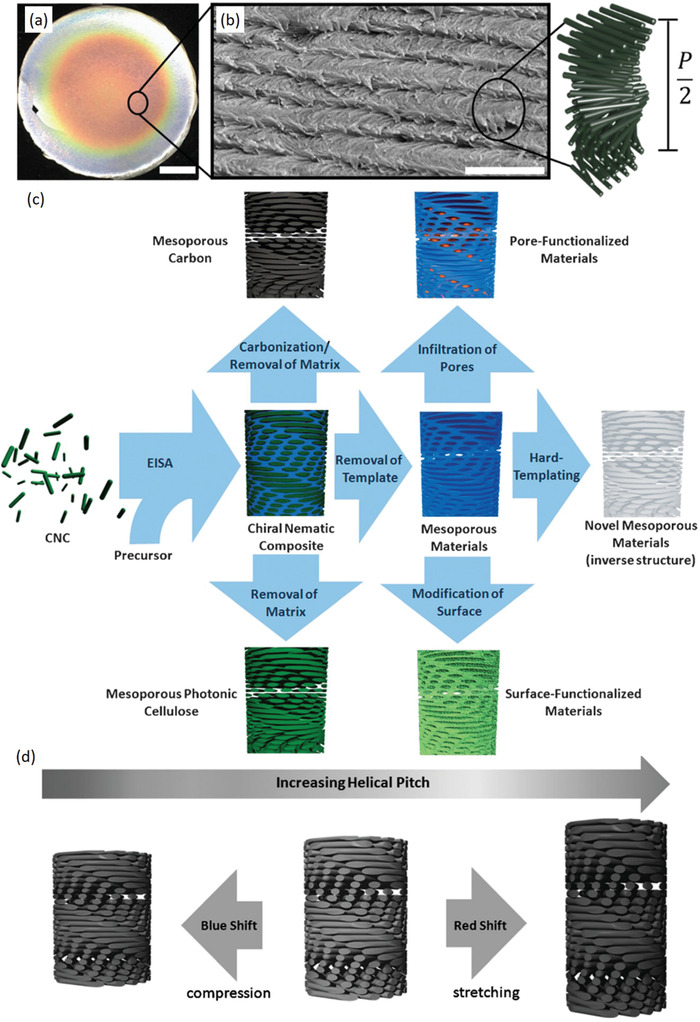
a) Optical and b) SEM images of a typical CNC film prepared through EISA, forming a chiral nematic helical structure. c) Schematic of the various synthetic routes to prepare functional materials with chiral nematic order based on the EISA process. d) Post‐synthetic manipulation of the chiral nematic pitch results in changes in the film optical properties, which can be useful in a variety of sensing applications. a,b) Adapted with permission.^[^ ^95^ ^]^ Copyright 2017, American Chemical Society. c,d) Adapted with permission.^[^ ^113^ ^]^ Copyright 2015, Wiley‐VCH.

Comprehensive work in the MacLachlan lab has demonstrated the potential to incorporate both hydrophilic and hydrophobic polymers into chiral nematic CNC films by either dispersing them in water,^[^ ^108^, ^109^ ^]^ polar organic solvents,^[^ ^110^ ^]^ or a mixture of both.^[^ ^111^ ^]^ Additionally, either the polymer matrix or the CNC template^[^ ^112^ ^]^ may then be selectively removed to yield mesoporous materials with chiral nematic structure (**Figure** **5**).^[^ ^113^ ^]^ This technique is extremely versatile, as material properties are readily tailored by manipulating variables including polymer type, drying conditions, post‐processing, and subsequent loading of new materials.^[^ ^113^, ^114^ ^]^ For example, by incorporating phenol formaldehyde resins into the precursor mixture, mesoporous photonic films were prepared which can swell in polar organic solvents, resulting in a red‐shift in the structural color of the film.^[^ ^115^ ^]^ By selectively crosslinking areas of the film via post‐processing in acid or formaldehyde, the swelling extent can be readily controlled, resulting in the appearance of latent images upon swelling.^[^ ^115^ ^]^ Furthermore, using a layer‐by‐layer fabrication technique, multiple thin films with different compositions/pitch lengths can be stacked to create complex structures with Janus‐type swelling useful as actuators for soft robotics applications.^[^ ^116^ ^]^ Recent work has also demonstrated the use of shear forces to unwind the CNC chiral nematic pitch within elastomeric composites; as the films are stretched, the birefringence of the film changes, presenting an opportunity to use these materials as colorimetric strain sensors.^[^ ^117^ ^]^

As previously discussed in Section 2.1, CNCs above the concentration for chiral nematic assembly are oriented with their helical chiral nematic director parallel to an applied magnetic field,^[^ ^118^, ^119^ ^]^ which over time can lead to a uniform chiral nematic texture.^[^ ^48^, ^120^, ^121^ ^]^ The kinetics of this process are readily controlled by varying the applied field strength.^[^ ^122^ ^]^ Interestingly, Frka‐Petesic et al. demonstrated the use of a magnetic field during the EISA process to control the self‐assembly, with the field direction dictating the orientation of the resulting chiral nematic pitch.^[^ ^123^ ^]^ Finally, it should also be mentioned that both electric fields^[^ ^124^ ^]^ and rotating magnetic fields^[^ ^125^, ^126^ ^]^ have been used to “unwind” the CNC chiral nematic pitch, resulting in eventual nematic alignment. Notably, Tatsumi et al. prepared anisotropic composites by mixing CNCs with hydroxyethylmethacrylate in the presence of a rotating magnetic field; subsequent photopolymerization enabled “locking” of the CNC orientation, which dictated the (anisotropic) mechanical properties of the composites.^[^ ^125^ ^]^

## 3 Material Processing‐Induced Alignment

In addition to the alignment of nanoscale building blocks, specific processing routes/assembly pathways can have a large influence on the resulting material macrostructure, properties, and functionalities.^[^ ^127^ ^]^ Several prominent examples are readily observed in nature, such as in bone formation, where porosity plays a key role in determining toughness, and in bamboo growth, where the sophisticated arrangement of fibrils into gradients leads to certain areas having high flexibility and others having structural rigidity within the same shoot.^[^ ^128^, ^129^ ^]^ These types of assemblies can lead to materials with enhanced properties or even novel functionalities which could not otherwise be achieved in unstructured materials comprised of identical chemical components. Therefore, the development of effective fabrication techniques for the creation of synthetic materials capable of emulating some of these forms and functions found in natural materials is of high interest. While numerous fabrication and processing techniques exist, with each designed for specific material application, here we focus on techniques which are relevant for the synthesis of nanocellulose‐containing materials with porous structures, such as foams and aerogels, and fibrous structures, such as spun filaments and 3D‐printed scaffolds; examples that have gathered increasing attention lately and have inherent structural properties that are intimately linked to their functionality.

### 3.1 Porous Structure

Lightweight cellular‐structured monoliths in the form of foams or aerogels are burgeoning as high‐performance structural and functional materials due to their low density, high porosity, large specific surface, high strength‐to‐weight ratio, and tunable porous architectures.^[^ ^130^, ^131^, ^132^ ^]^ Research interest into the use of nanocellulose in particular for developing such materials is growing rapidly, due in part to their tunable surface chemistries, stabilizing characteristics, and outstanding mechanical properties, in addition to possessing several of the other desirable features of foams and aerogels.^[^ ^2^, ^3^, ^5^, ^132^, ^133^, ^134^, ^135^, ^136^, ^137^, ^138^, ^139^, ^140^ ^]^ Typically, nanocellulose foams/aerogels are prepared from either a dispersion or hydrogel, which is subsequently dried via convective, super‐critical, or freezing‐based methods.^[^ ^132^ ^]^ In this section, we focus on the intrinsic properties of nanocellulose allowing for the bottom‐up fabrication of structured monoliths with advanced functionality: the ability to stabilize interfaces/other nanoparticles, the ability to introduce structural anisotropy, and the ability to act as a template for other materials.

#### 3.1.1 Stabilizing Composite Materials

There has been a growing interest in the rational design and controlled assembly of composite foams and aerogels based on various functional nanomaterials, owning outstanding mechanical, electrical, thermal, or optical properties; such materials are sought after for diverse applications such as energy storage, catalysis, pressure/strain sensing, oil/water separation, thermal insulation, electromagnetic interference shielding, and flexible devices.^[^ ^5^, ^133^, ^139^, ^140^ ^]^ However, several of these functional nanomaterials have a strong tendency to aggregate and stack together, resulting in inferior properties of the assembled composites, and hindering their full potential. Conversely, due to both the inherent crystal structure of nanocellulose (its sheet‐like structure exposes more hydrophilic edges in plane with the sugar ring compared to in the perpendicular direction, resulting in molecular amphiphilicity)^[^ ^141^, ^142^ ^]^ and its abundant hydrophilic polar surface groups (i.e., —OH and —COOH), nanocellulose forms a stable aqueous dispersion with a highly negative Zeta potential.^[^ ^140^ ^]^ In turn, this allows nanocellulose to act as a green, stable, and low‐cost dispersant, enabling homogenous mixing of CNC/CNF and other nanomaterials, such as carbon quantum dots,^[^ ^143^ ^]^ magnetic nanoparticles,^[^ ^144^ ^]^ CNTs,^[^ ^145^, ^146^, ^147^, ^148^, ^149^, ^150^, ^151^, ^152^ ^]^ graphene,^[^ ^149^, ^153^, ^154^, ^155^, ^156^ ^]^ boron nitride (BN),^[^ ^157^, ^158^, ^159^ ^]^ and molybdenum disulfide (MoS_2_),^[^ ^160^ ^]^ without the use of additional surfactants or chemical functionalization.

**Figure 6:**
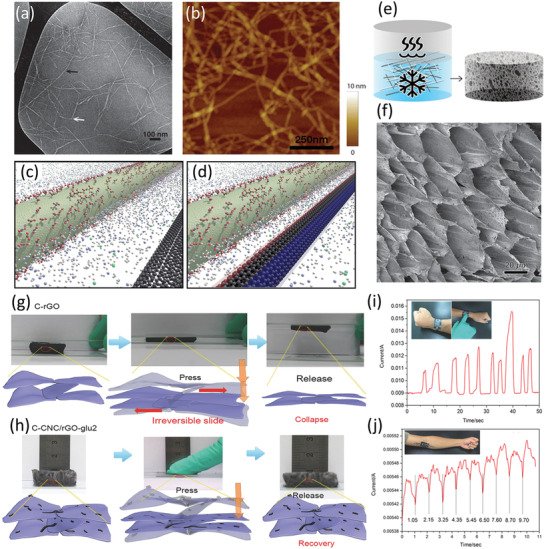
a) TEM and b) AFM images of CNF/SWCNT dispersions dried on a silicon wafer. c) Schematic depiction of the interaction between a charged nanocellulose rod (green) and an SWCNT (black) in water. d) As the nanoparticles approach each other, a dipole is induced on the SWCNT surface. e) Schematic of the preparation of CNF/SWNT aerogels by freezing and subsequent freeze‐drying, and f) SEM image of the aerogel containing 12 wt% SWCNT. g) Schematic microstructures, elasticity mechanisms, and digital photographs of the compression and collapse of rGO within CNC/rGO aerogels. h) Wearable device assembled from CNC/rGO aerogels for human blood pressure detecting, demonstrating: i) the current signal caused by finger clicks, and j) pressure sensing capability for biomonitoring. a,b,e,f) Adapted with permission.^[^ ^155^ ^]^ Copyright 2014, American Chemical Society. c,d) Adapted with permission.^[^ ^149^ ^]^ Copyright 2017, American Chemical Society. g–j) Adapted with permission.^[^ ^161^ ^]^ Copyright 2018, Wiley‐VCH.

In order to achieve the best possible mechanical and electrical properties of CNT assemblies, a high‐quality dispersion is necessary to separate the CNT bundles into stable colloid particles. Typically, this is only achievable through modification of the CNTs themselves, however in practice this can substantially increase the cost of CNT and can also disrupt the CNT microstructure, leading to a loss of mechanical strength and lower electrical conductivity, as well as some environmental contamination. In contrast, nanocellulose can act as an excellent aqueous dispersing agent for as‐prepared single‐walled CNTs (SWCNT), making possible low‐cost exfoliation and purification of SWCNTs.^[^ ^145^, ^155^ ^]^ Hamedi et al. reported CNF‐assisted aqueous dispersions of SWCNTs as a precursor for highly conducting and strong nanostructured composites thereof including the aerogels.^[^ ^155^ ^]^ Transmission electron microscopy (TEM) and atomic force microscopy (AFM) images of the CNF/SWCNT fibers dried from the dispersed state show individualized nanofibers having diameters of less than 10 nm, corresponding well with individual or small bundles of either CNF or SWCNTs (**Figure** **6**a,b). Hajian et al. studied the influences of nanocellulose surface charge density on the quality and quantity of CNTs in aqueous media,^[^ ^149^ ^]^ arguing that the stabilization of carbon nanomaterials including CNTs and reduced GO (rGO) in water was mainly attributed to the association between carbon nanomaterials and nanocellulose. The authors speculated that this association resulted from fluctuation of the counterions on the nanocellulose surface inducing dipoles in the sp^2^ carbon lattice surface on the carbon nanomaterial surface (Figure 6c,d). Moreover, the existence of surface charges on nanocellulose leads to electrostatic stabilization of the nanocellulose–carbon nanomaterial complexes, effectively preventing aggregation of the nanomaterials. Based on this understanding, nanocellulose with a high surface charge density (1400 μequiv g^−1^) was used to disperse and stabilize carbon nanomaterials in water increasing the dispersion limit of CNTs up to 75 wt%, compared to a CNT dispersion limit of 10 wt% by using nanocellulose with a low charge density (290 μequiv g^−1^). Chauvet et al. reported that CNCs are similarly able to disperse high amounts of CNTs (both single‐ and multi‐walled) in water.^[^ ^147^, ^152^ ^]^ These experimental results and theoretical considerations provide support that nanocellulose can be used as an efficient dispersing agent in the assembly of advanced composites including lightweight and conducting CNF/SWCNT aerogels (Figure 6e,f).^[^ ^155^ ^]^ In this work, the CNF/SWCNT aerogels showed a conductivity of 0.014 S m^−1^ at an SWCNT content of 12 wt%. Finally, Xu et al. effectively utilized CNF to disperse CNT in water at CNT mass ratios ranging from 10 to 70 wt%.^[^ ^151^ ^]^ Self‐assembled cellular foam monoliths from the CNF/CNT dispersions were achieved with ultralow density (9.2 mg cm^−3^), strong fatigue resistance, and ultrabroad‐band microwave absorption performance.

The exfoliation/dispersion of 2D nanomaterials is of great importance due to their unique properties and applications in electronics and other functional devices. Liquid exfoliating/dispersing 2D materials in a scalable and environmentally friendly way is quite attractive since greener solvents and dispersants can enable cost‐effective large‐scale production processes that minimize or eliminate the need for properly disposing potentially harmful chemicals. Hu et al. successfully used TEMPO‐oxidized CNF as a green dispersant to disperse 2D BN and MoS_2_ in water.^[^ ^160^ ^]^ They proposed the mechanism for the dispersion of 2D materials by CNF: CNF attaches to the flakes through the interaction between its hydrophobic sites and the flakes hydrophobic plane as well as hydrogen bonding between the CNF hydroxyl groups and the defective edges of the 2D materials. The flakes were stabilized due to steric hindrance and the electrostatic repulsive forces generated by the charged CNF carboxyl groups. Xiong et al. reported an efficient and universal amphiphilicity‐driven assembly strategy to construct “hairy” flexible hybrid nanosheets with a 1D CNF net conformally wrapped around 2D GO monolayers.^[^ ^153^ ^]^ By tailoring the surface chemistry of GO sheets, the CNFs could either tightly surround the individual sheets or separate from the GO surfaces. This could be attributed to the competition among the electrostatic repulsive interactions, the hydrophobic–hydrophobic interactions, and hydrogen bonding between the CNFs and GO sheets. This behavior is considered to offer efficient assistance in the dispersion or exfoliation of 2D nanomaterials. Importantly, despite some work about the liquid exfoliation or dispersion of 2D graphene through the employment of nanocellulose being reported,^[^ ^147^, ^154^ ^]^ the construction of effective functional cellular 3D macrostructures remains challenging.

In addition to acting as an effective dispersant, the high specific surface area, excellent mechanical properties, large aspect ratio, and abundant functional groups of nanocellulose render the possibility to bind or network with otherwise weakly interacting nanomaterials,^[^ ^162^, ^163^, ^164^, ^165^ ^]^ which in turn benefits the structural stability and functional attributes of the resulting cellular monoliths.^[^ ^131^, ^161^, ^166^, ^167^ ^]^ Zhong et al. utilized CNC to effectively enhance the interaction between rGO layers, leading to an ultralight, flexible, and superstable lamellar carbon aerogel;^[^ ^161^ ^]^ insufficient interaction among rGO layers led to irreversible movement during compression, while the carbonization of CNC created a strong interaction (carbon welding) among rGOs to form flexible layers with reversible movement, thus resulting in a stable and elastic structure (Figure 6g,h). Furthermore, CNCs also acted as an essential nanosupport to reduce deformation and shrinkage of rGO aerogels during freeze‐drying and pyrolysis. Finally, the aerogel demonstrated promising application in the form of wearable devices (Figure 6i,j). The authors continued to utilize CNC as a nanobinder and nanosupport to connect MXene nanosheets,^[^ ^162^, ^166^ ^]^ which are not easily assembled into robust cellular architectures because of their weak gelation ability and strong tendency to agglomerate. Abundant H, O, and F atoms in MXene sheets led to strong hydrogen bond formation between MXene and CNCs, and were able to connect MXene sheets together into an aerogel with enhanced mechanical strength. These aerogels could undergo an extremely high compression strain of 95% and long‐term compression and display both extremely high sensitivity (114.6 kPa^−1^) and wide linear range (50 Pa–10 kPa) as electromechanical pressure sensors. Hu et al. also proposed CNC as a nanoreinforcement/nanosupport to fabricate a robust chitosan/CNC aerogel, showing superior structural stability, high compressibility, and elasticity at 95% strain.^[^ ^167^ ^]^

#### 3.1.2 Structure‐Directing Materials

**Figure 7:**
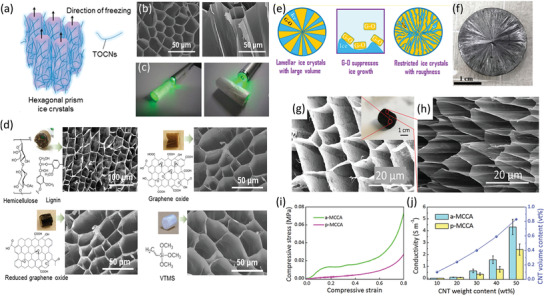
a) Schematic diagram of the preparation of monoliths with microhoneycomb structure via FD, illustrating the unidirectional ice‐crystal growth during the freezing step. b) SEM images of the cross‐section (left) and longitudinal section (right) of the resulting CNF monoliths, with c) laser penetration tests demonstrating their anisotropic properties. d) Optical and SEM images of a variety of composite monoliths with different chemical compositions, demonstrating the consistent formation of microhoneycomb‐like structure. e) Illustration of the radiating structure formed by ice crystal templating in bidirectional FD of GO‐based aerogels, and f) optical image of GO/CNF nanocomposite aerogel with this radiating structure. g) Cross‐sectional SEM image of CNF/CNT aerogel, observed in a direction perpendicular to the microchannels, and h) SEM image of the cross‐section of the same aerogel in a direction parallel to the microchannels. i) Compressive stress–strain curves of the aerogel in both directions, and j) electric conductivities in both directions of the aerogels with different CNT weight contents. a–d) Adapted with permission.^[^ ^184^ ^]^ Copyright 2016, American Chemical Society. e,f) Adapted with permission.^[^ ^185^ ^]^ Copyright 2018, American Chemical Society. g–j) Adapted with permission.^[^ ^180^ ^]^ Copyright 2019, Wiley‐VCH.

Most cellular structures are isotropic with random cross‐linked structures rather than having ordered controllable morphologies,^[^ ^135^, ^168^, ^169^, ^170^, ^171^, ^172^, ^173^, ^174^, ^175^, ^176^, ^177^, ^178^, ^179^ ^]^ which fundamentally limits their application in areas where anisotropy is desired/beneficial, such as directional mass transport in filtration/separation, tissue engineering of oriented tissues, and directional electrical/thermal conduction/insulation.^[^ ^180^, ^181^ ^]^ There are several reports demonstrating that both CNF and CNC can be assembled into xylem‐like anisotropic monoliths (typically with characteristic microhoneycomb‐like structures) through simple freeze‐drying (FD) or freeze‐casting processes.^[^ ^3^, ^182^, ^183^ ^]^ Yang et al. demonstrated the strong propensity of CNF toward forming a microhoneycomb structure through FD due to the CNF nanofiber morphology, indicating that the microstructure formed through FD highly depends on the chemical/physical properties of the precursor solution (**Figure** **7**a–c).^[^ ^184^ ^]^ This strong tendency of CNF to form microhoneycomb monoliths through FD was extensively investigated via surface grafting or by mixing a combination of additional components, including polyurethane, lignin, GO, and vinyltrimethoxysilane (Figure 7d). For example, even using only 20 wt% CNF in a CNF/polyurethane precursor sol, a microscopic honeycomb monolith can still be obtained.^[^ ^184^ ^]^ Wang et al. reported the assembly of GO into a vertical and radially aligned cellular structure via a bidirectional FD approach.^[^ ^185^ ^]^ In this work, they found that “constraints” imposed by the GO sheets led to the decreased volume irregular shape changes of each ice crystal, resulting in the irregular cell wall/porous structure in the GO cellular monoliths. By incorporating CNF with abundant hydroxyl groups, the group was able to decrease the interaction between GO and ice but increase the CNF/ice and CNF/GO interaction, resulting in a regular and well‐ordered cell wall/porous structure in the assembled anisotropic aerogels. Wicklein et al. reported monoliths consisting of CNF, GO, sepiolite nanorods, and boric acid with microhoneycomb structure.^[^ ^186^ ^]^ Through simple FD of this mixed suspension, super‐insulating, fire‐retardant, and ultralight yet robust anisotropic foams with aligned channels were produced, which performed better than traditional polymer‐based insulating materials. These anisotropic microhoneycomb monoliths showed pores having a diameter of ≈20 μm and a cell wall thickness of 0.2–0.4 μm, and demonstrated both anisotropic thermal conductivities and mechanical properties.^[^ ^186^ ^]^

By utilizing the unique structure‐directing function of nanocellulose, a variety of anisotropic monoliths with ordered cell walls and well‐designed chemical compositions has been obtained.^[^ ^151^, ^180^, ^184^, ^186^, ^187^, ^188^, ^189^, ^190^ ^]^ This has thus enabled the fabrication of function‐targeted cellular monoliths with unidirectional pores/microchannels. For example, Wang et al. reported the preparation of a CNF/CNT composite anisotropic microhoneycomb hybrid aerogel with unidirectional aligned penetrating microchannels, designed to be anisotropic in compressive properties and electrical conductivity (Figure 7g–j).^[^ ^180^ ^]^ The aerogels were additionally used as a filler material for a composite with polydimethylsiloxane, resulting in a material with good mechanical and electrical properties with clear directionality for use as a directional strain sensor. Mi et al. fabricated highly compressible, ultralight anisotropic CNF/graphene aerogels by bidirectional FD for selective oil absorption.^[^ ^191^ ^]^ Work by Chau et al. demonstrated that by varying the ratio and overall amount of CNC within CNC‐POEGMA aerogels, the pore morphology could be tuned from random to anisotropic (columnar and lamellar).^[^ ^192^ ^]^ The aerogels also displayed anisotropic swelling and mechanical performance as a direct result of the pore morphology obtained.

#### 3.1.3 Templating Materials

Due to the excellent mechanical properties and abundant surface functional groups on nanocellulose, the fabrication of robust biopolymer/nanocellulose composites with high porosity can be formed, using the CNF as both a structural and chemical template.^[^ ^171^, ^193^, ^194^ ^]^ Such aerogels have broad potential, as the nanocellulose can be easily integrated (embedded or loaded) with a variety of materials, including conductive polymers,^[^ ^195^, ^196^ ^]^ CNTs,^[^ ^188^, ^197^, ^198^, ^199^, ^200^ ^]^ and graphene.^[^ ^201^, ^202^, ^203^ ^]^ Carlsson et al. presented conductive CNF aerogels via chemically polymerizing pyrrole onto TEMPO‐oxidized CNF dispersed in water, followed by different drying methods, including supercritical CO_2_ drying, freeze drying, and ambient drying.^[^ ^195^ ^]^ Wang et al. incorporated CNT into a CNF suspension to form aerogels via freeze drying, resulting in a hybrid material with increased ductility, reduced brittleness, and electroactivity.^[^ ^197^ ^]^ Zheng et al. and Gao et al. both reported highly flexible, all‐solid‐state supercapacitors that use CNF/rGO/CNT and CNF/rGO hybrid aerogels as electrodes, respectively.^[^ ^188^, ^202^ ^]^ Because of the porous structure of these hybrid aerogel electrodes and the excellent electrolyte absorption properties of CNF, the resulting flexible supercapacitors exhibited a high specific capacitance and a remarkable cycle stability.

**Figure 8:**
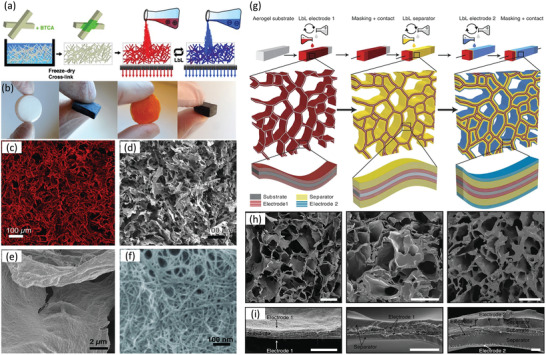
a) Schematic depiction of the cross‐linking, fabrication strategy, and LbL assembly technique for CNF aerogels. b) Optical images of LbL‐functionalized aerogels in the dry state during various stages of fabrication, c) 3D reconstruction obtained from confocal microscopy slices of a CNF template aerogel in the dry state, and d–f) corresponding cross‐section SEM images showing the different hierarchical structures present within the composite aerogels. g) Schematic of the LbL process used to assemble 3D CNF aerogel‐based supercapacitors, and h,i) corresponding cross‐section SEM images of the device at various stages of assembly. Scale bars are: h) 50 and i) 2 μm. a–f) Adapted with permission.^[^ ^209^ ^]^ Copyright 2014, Wiley‐VCH. g–i) Adapted with permission.^[^ ^210^ ^]^ Copyright 2015, Springer Nature.

Although nanocellulose‐based aerogels can be readily produced without additional crosslinking due to hydrogen bond formation between fibers, these hydrogen bonds are readily broken upon submersion in water, which can severely limit the practical application of such materials.^[^ ^132^ ^]^ Therefore, covalent crosslinking represents an interesting solution to increase the robustness of nanocellulose‐based aerogels. As such, several different crosslinking techniques have been explored for enhancing the strength and wet‐integrity of porous nanocellulose scaffolds.^[^ ^135^, ^204^, ^205^ ^]^ Kobayashi et al. demonstrated the preparation of CNF aerogels through acid‐induced gelation,^[^ ^172^ ^]^ and Zhang et al. accomplished covalent cross‐linking of CNF aerogels by utilizing polyamide‐epichlorohydrin resin as crosslinker, leading to improved shape recovery in water.^[^ ^204^ ^]^ Jiang and Hsieh prepared CNF aerogels using a chemical crosslinking approach where the hydrogels were solvent exchanged with acetone and then cross‐linked with methylene diphenyl diisocyanate.^[^ ^206^ ^]^ Hamedi et al. applied BTCA^[^ ^207^, ^208^ ^]^ as a cross‐linker to form ester linkages with cellulosic hydroxyl groups, leading to cross‐linked CNF aerogels with a porosity close to 99%, high strength, and shape integrity in water.^[^ ^209^ ^]^ Here, the high surface charge of the nanocellulose further imparted the possibility for noncovalent yet strong ionic interactions with other functional polymers or nanomaterials. As a result, Hamedi et al. went on to demonstrate cross‐linked wet‐stable CNF aerogels as an ideal template for the layer by layer (LbL) assembly of thin films of biomolecules, conducting polymers, and CNT (**Figure** **8**a–f). Using this same LbL assembly technique, the group further demonstrated 3D energy storage devices based on the self‐assembly of interdigitated thin films on the surface of an open‐cell CNF aerogel substrate (Figure 8g–i).^[^ ^210^ ^]^ The results effectively show that LbL self‐assembly on nanocellulose aerogels is a rapid, precise, and scalable fabrication route for building high surface area 3D thin‐film devices.

Although impressive progress has already been achieved in the fabrication of advanced materials using CNF aerogels as supports, the structural brittleness of CNC aerogels, resulting from the rigidity of CNCs themselves and the limited mobility/entanglements between individual CNCs, is a crucial challenge for realizing the mechanical performance necessary in practical applications.^[^ ^135^ ^]^ As such, physical crosslinking strategies do not work as well for CNC aerogels as compared to CNF aerogels, and therefore chemical cross‐linking is far more effective for improving the mechanical performance of CNC aerogels.^[^ ^211^, ^212^, ^213^ ^]^ Yang and Cranston demonstrated the first chemically cross‐linked CNC aerogels based on hydrazone cross‐linking of hydrazide and aldehyde‐functionalized CNCs.^[^ ^214^ ^]^ The resulting aerogels were ultralightweight (5.6 mg cm^−3^) and highly porous (99.6%) with a bimodal pore distribution (mesopores < 50 nm and macropores > 1 μm). These cross‐linked CNC aerogels showed tailorable mechanical performance and shape recovery abilities in both air and liquid. Many subsequent strategies based on chemical cross‐linking of CNCs have thus been developed to fabricate CNC aerogels combined with other organic or inorganic components.^[^ ^215^, ^216^, ^217^, ^218^, ^219^, ^220^, ^221^ ^]^ The Cranston group went on to further demonstrate aerogels combining CNCs with various capacitive nanoparticles including polypyrrole nanofibers, CNT, spherical manganese dioxide nanoparticles, and metal‐organic frameworks (MOFs), leading to lightweight and freestanding devices with enhanced performance.^[^ ^222^, ^223^ ^]^ Finally, the same group also combined functional MOFs into flexible and porous CNC aerogels through a straightforward sol–gel process, followed by freeze drying.^[^ ^224^ ^]^ The CNCs alone formed colloidally stable suspensions but assembled into covalently crosslinked clusters with entrapped MOFs, when mixed together. Importantly, the MOFs retained their crystallinity, porosity, and accessibility when entrapped within the aerogels, making them ideal absorbents for water purification and other separations applications. Matsumoto and Kitaoka demonstrated similar results by successfully embedding MOFs into nanoporous CNF films, showing ultraselective gas permeance to CO_2_ in mixtures with CH_4_.^[^ ^225^ ^]^ Together, this work effectively demonstrates that CNC aerogels can act as universal substrates or matrices for a large range of nanosized materials.

### 3.2 Fibrous Structure

In parallel with highly porous materials, filamentous/fibrous nanocellulose‐based materials have also gained significant research interest due to their enhanced mechanical properties, stability, facile production strategies, and overall versatility.^[^ ^39^, ^65^, ^226^ ^]^ Techniques such as wet‐spinning, dry‐spinning, electrospinning, and 3D printing have all been successfully used to prepare nanocellulose fibers/filaments, which in turn can be organized into woven materials, porous substrates, and even structurally precise scaffolds for tissue engineering. As both CNCs and CNFs present shear‐thinning properties, they are naturally well‐suited to be used in fiber spinning/extrusion processes, albeit each typically for different reasons; the longer and more flexible CNF can be used in a pure suspension to form stable fibers due to enhanced nanofibril entanglement, while the shorter and more rigid CNC can be used as a viscosity modifier/mechanical property enhancer within composite fibrous materials.^[^ ^11^ ^]^ In this section, we focus on the use of nanocellulose in the bottom‐up fabrication of fibrous/filamentous materials through both spinning and printing/extrusion‐based techniques, highlighting the specific properties and methods used to prepare advanced materials with unique properties.

#### 3.2.1 Materials from Spun Fibers

**Figure 9:**
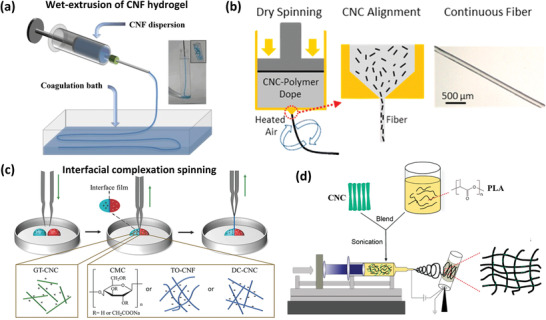
Various techniques used in the creation of nanocellulose filaments. a) Preparation of CNF filaments via wet extrusion into a coagulation bath. b) Schematic dry spinning process for CA/CNC composite filaments. c) Drawing process for preparing nanocellulose‐based filaments by interfacial complexation of oppositely charged nanocellulose suspensions. d) Preparation of PLA/CNC fibrous mats via electrospinning. a) Adapted with permission.^[^ ^228^ ^]^ Copyright 2011, Wiley‐VCH. b) Adapted with permission.^[^ ^56^ ^]^ Copyright 2014, American Chemical Society. c) Adapted with permission.^[^ ^229^ ^]^ Copyright 2018, Wiley‐VCH. d) Adapted with permission.^[^ ^230^ ^]^ Copyright 2018, American Chemical Society.

Spun fibers are commonly manufactured for a wide variety of uses such as filters, textiles, and composites, and due to the relative simplicity of spinning techniques, there has been plenty of literature investigating the spinning of fibers from several natural sources including plant fibers, cellulose solutions, and nanocellulose suspensions.^[^ ^227^ ^]^ Spinning has been successfully used to create both neat nanocellulose (albeit only with CNF due to lack of entanglement/spinnability for CNC) and nanocellulose‐composite filaments, the diameter of which can be controlled from several hundred nanometers to several hundred micrometers, depending on the detailed spinning technique and parameters used (**Figure** **9**).^[^ ^168^ ^]^ In terms of spinning technique, typically wet‐, dry‐, and electrospinning are used to prepare fibers with different properties, while in terms of spinning parameters, temperature, draw ratio, the use of a coagulant, drying technique, concentration, and the presence of additives can be readily controlled to further tune the filament properties.^[^ ^39^ ^]^ Regardless of technique, in spinning, the precursor material is extruded through a forming element, after which solidification is achieved through facile solvent evaporation (dry spinning), or precipitation in a coagulant/antisolvent (wet spinning). Importantly, although there has been some research investigating the alignment of individual CNC/CNF during various spinning/extrusion processes, this section focuses on the investigation of material properties arising mainly from the nature of the filaments themselves, and not as a result of nanocellulose alignment.

Dry spinning is a low‐cost and environmentally friendly method for producing filaments directly from an aqueous suspension of CNF. However, a certain CNF concentration must be reached in order to form a continuous filament; Hooshmand et al. identified this concentration as 6.5 wt% for their CNF suspension, also noting that as concentration is further increased, both the potential for CNF orientation during spinning and the resulting mechanical properties of the formed filaments decrease.^[^ ^231^ ^]^ In another study, Ghasemi et al. investigated the effects of drying temperature on CNF filaments, and determined that this did not significantly influence the mechanical properties of filaments, suggesting that higher temperatures could safely be used to speed up the drying process.^[^ ^232^ ^]^ Hybrid monofilaments of poly(acrylic acid) (PAA) and 1,6‐hexanediol diglycidyl ether (16DGE), compounded with CNF and graphene, were thermally cross‐linked and subsequently spun from aqueous solution via dry‐spinning.^[^ ^233^ ^]^ The obtained filament was elastic and flexible because of the cross‐linking between PAA and 16DGE, among where the CNF acted as reinforcing nanofiller. In another similar work, CNC was also used as reinforcements of CA filaments via dry‐spinning, where maximum improvements of 137% in tensile strength and 637% in elastic modulus were obtained.^[^ ^56^ ^]^ The addition of hydroxyethyl cellulose in CNF filaments via dry‐spinning was shown to improve mechanical properties of these bio‐based filaments as well as their compatibility with epoxy.^[^ ^234^ ^]^

Due to the presence of a coagulant acting to enhance fiber gelation and entanglement, wet spinning of lower concentration neat CNF filaments is possible versus dry‐spinning (the use of a flow‐focusing set‐up can further decrease the CNF concentration required to form continuous filaments^[^ ^64^ ^]^).^[^ ^39^ ^]^ Furthermore, and similar to dry‐spun CNF filaments, in wet‐spun CNF filaments using CNF with higher surface charge (typically achieved via TEMPO‐mediated oxidation or carboxymethylation), larger aspect ratio (related to CNF source and processing conditions) and lower overall CNF concentration (above the limit of spinnability) leads to improved mechanical performance of the resulting filaments.^[^ ^39^ ^]^ Albeit, without appropriate crosslinking, mechanical performance deteriorates drastically in the presence of water.^[^ ^38^ ^]^ To combat this fact, Geng et al. demonstrated that wet‐spun CNF filaments physically cross‐linked in an acetone coagulation and subsequently chemically cross‐linking using a polyamide‐epichlorohydrin resin showed improved strength and modulus versus uncrosslinked filaments (369.8 MPa and 28.9 GPa vs 268.7 MPa and 22.8 GPa, respectively).^[^ ^235^ ^]^ Vuoriluoto et al. demonstrated UV crosslinking of benzophenone/CNF filaments, which displayed improved wet strength over un‐cross‐linked fibers.^[^ ^236^ ^]^ Walther et al. demonstrated mechanically robust CNF filaments by post‐modifying wet‐spun filaments.^[^ ^228^ ^]^ Furthermore, electrical conductivity was also achieved through this same process, whereby CNF filaments were dipped into a toluene solution containing dodecyl benzene sulfonic acid‐doped polyaniline, which was shown to tightly adsorbs onto and into the pores of the CNF filaments. The effects of post‐modification of wet‐spun CNF filaments via chemical vapor deposition have also been investigated; the surface conformation of the adsorbed species (here, a continuous layer or hairy/brush‐like assembly) significantly affected the resulting filaments interaction with water.^[^ ^237^ ^]^

There has been extensive research looking at the formation of nanocellulose‐based composite filaments via wet‐spinning in order to incorporate additional functionality into the resulting fibers. Richardson et al. demonstrated the preparation of CNC/MOF composite fibers via direct mixing and subsequent extrusion, whereby the presence of MOF acts to both aid in crosslinking of the CNC and add additional functionality to the fibers.^[^ ^238^ ^]^ Wan et al. demonstrated that conductive CNF/SWCNT composite filaments obtained through wet‐spinning with high strength (up to ≈472.17 MPa) could readily be prepared,^[^ ^145^ ^]^ while Wang et al. directly carbonized lignin/CNF filaments at 900 °C to also obtain filaments with electrical conductivity (as high as 103 S cm^−1^).^[^ ^239^ ^]^ In addition to incorporating materials with added functionality, there has been some research into the use of preparing composite filaments with a core/shell configuration through coaxial spinning.^[^ ^62^ ^]^ In this technique, control over the interfacial interactions is possible by carefully selecting both the core/shell materials, and the coagulant; Lundahl et al. showed that by using CNF as the core material and either guar gum (GG) or CA as the shell material, the resulting filament shell displayed different levels of interactions with the CNF core upon coagulation in various solvents (acetone, ethanol, or water).^[^ ^62^, ^240^ ^]^ Finally, CNC/PVA composite filaments have also been produced via coaxial coagulation spinning, whereby the presence of CNCs provided a direct reinforcing effect, leading to enhanced strength and stiffness of filaments.^[^ ^241^ ^]^

Interfacial complexation (IC) spinning/drawing is a unique technique based on the spontaneous filament self‐assembly upon drawing two insoluble and oppositely charged polyelectrolyte complexes at an interface.^[^ ^242^ ^]^ This technique has been applied to fabricate continuous nanocellulose‐based filaments under aqueous conditions, whereby cationic CNC and an anionic cellulose material (sodium carboxymethyl cellulose, TEMPO‐oxidized CNF, or dicarboxylated CNCs) were used to successfully prepare continuous filaments.^[^ ^229^ ^]^ In a similar example, Reid et al. demonstrated the preparation of CNC‐polyamide janus nanocomposite fibers via one‐step interfacial polymerization, whereby CNCs were mixed in the aqueous phase with diamine monomers, opposite acyl chloride monomers in an oil phase; in addition to filament formation, this method could be used to form films or capsules via the same mechanism, depending on the mixing technique employed.^[^ ^243^ ^]^ Finally, Nechyporchuk et al. prepared composite filaments with a CNF core and a silica nanoparticle shell via wet‐spinning of anionic CNF suspensions into an acidic coagulation bath containing cationic SNP; the SNP shell imbued the composite filaments with enhanced flame retardency, attributed to the capacity of SNP to promote char formation and heat insulation on the fiber surface.^[^ ^244^ ^]^

Another widely reported method for producing filaments/fibers is electrospinning, which acts to draw a polymer solution via the influence of an applied electric field.^[^ ^226^ ^]^ There have been many studies focused on the preparation of electrospun filaments containing nanocellulose, of which the majority use CNC as a reinforcing agent for polymer‐based composite nanofibers.^[^ ^230^, ^245^, ^246^, ^247^, ^248^ ^]^ Notably, Li et al. prepared composite nanofiber mats via coaxial electrospinning, whereby a mixture of poly(methyl methacrylate) and varying concentration of CNC was used as the shell fluid and polyacrylonitrile was used as the core fluid; here, increasing the CNC concentration significantly increased the conductivity of the spinning solution, favoring the formation of uniform fibers.^[^ ^249^ ^]^ Han et al. demonstrated a similar coaxial electrospinning technique to prepare conductive polyaniline‐coated nanofibers having a core of CNC and CNT, whereby CNC is used to stabilize CNT as discussed in the previous section; these conductive materials were used to prepare flexible supercapacitor electrodes.^[^ ^250^ ^]^ Finally, several research groups have studied the impact of electrospun mats with uniaxially aligned fibers, prepared using a rotating collector for directing cell growth and other biomedical applications;^[^ ^246^, ^251^, ^252^ ^]^ note that here, fiber alignment (and not individual nanocellulose particle alignment) is critical for creating the desired functionality.

#### 3.2.2 3D‐Printed Materials

**Figure 10:**
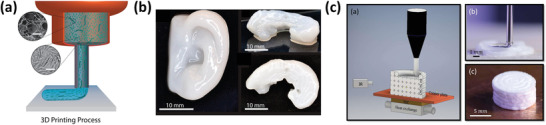
a) Schematic diagram of 3D‐printing process. b) Examples of complex structures produced via 3D printing; human ear (left), and side/top views of a sheep meniscus (right). c) Schematic illustration of the DCW process, and resulting aerogel. a) Adapted with permission.^[^ ^257^ ^]^ Copyright 2019, Wiley‐VCH. b) Adapted with permission.^[^ ^253^ ^]^ Copyright 2015, American Chemical Society. c) Adapted with permission.^[^ ^213^ ^]^ Copyright 2019, MDPI.

The use of 3D printing to form nanocellulose‐based scaffolds has gained significant research interest recently, particularly in the field of biomedical applications, due to the inherent gel forming properties/shear thinning behavior and general biocompatibility of nanocellulose as an ink. Furthermore, the versatility of the 3D‐printing process enables the formation of 3D structures with complex architectures (**Figure** **10**).^[^ ^65^ ^]^ Although there has been some work (discussed in Section 2 ^[^ ^66^, ^67^ ^]^) focusing on the alignment of nanocellulose during 3D printing, this section focuses on additional nanocellulose properties making it an interesting material for processing via 3D printing. As with the other methods discussed throughout this report, nanocellulose scaffolds prepared via 3D printing require some form of crosslinking for the formation of extruded filaments with good shape fidelity, as well as overall stability of the part (especially in aqueous conditions). One common crosslinking strategy employed is the ionic crosslinking of natural materials such as alginate or gelatin via submersion in a CaCl_2_ bath following printing; in this case, the nanocellulose component (either CNCs or CNFs) is typically incorporated due to its shear thinning properties to improve shape fidelity during the printing process.^[^ ^253^, ^254^, ^255^, ^256^ ^]^ Hausmann et al. demonstrated the wet densification of 3D‐printed CNC/CNF composite scaffolds via their submersion in differing solvents and subsequent polymerization of monomer species present in the solvent bath.^[^ ^257^ ^]^ By varying the CNC/CNF ink ratio along with the filament orientation with respect to the applied stress, scaffolds with drastically different mechanical properties could be achieved. Wang et al. demonstrated that surface modification of CNCs with photoinitiating bis(acyl)phosphane oxide groups enabled the polymerization of monofunctional methacrylate moieties during 3D printing, resulting in scaffolds with good shape persistence and improved mechanical properties.^[^ ^258^ ^]^

## 4 Future Outlook and Critical Challenges

In this report, we have discussed the recent progress in the bottom‐up fabrication of nanocellulose‐based materials. The breadth and quality of research accomplished to date indicates that the use of both CNC and CNF allows for the assembly of complex advanced materials with tailorable properties for a wide range of applications. By utilizing the unique properties of nanocellulose in pure suspension or within composites, improved biomimetic materials with controlled and hierarchical architectures are possible. The shape/diamagnetic anisotropy of individual nanocellulose particles enables their facile alignment/orientation within films, fibers, and gels, and can even be used to direct the orientation of other nanomaterials to create or enhance functionality.^[^ ^259^, ^260^ ^]^ Furthermore, their inherent surface charge/amphiphilicity affords the ability to effectively stabilize interfaces and prevent nanoparticle aggregation, allowing for the formation of robust porous monoliths in the form of foams and aerogels. Finally, their ability to act as a viscosity modifier/reinforcement agent enables the production of strong fibers, filaments, and structural templates for other functional materials. In general, materials showcasing these various types of nanocellulose structuring/ordering can lead to enhanced mechanical performance, interesting optical, electrical, thermal, biological, or anisotropic properties, and advanced biomimicry, which in turn represents a promising achievement for applications within a wide range of fields. However, in order to fully realize a realistic platform of practical nanocellulose‐based materials, there are several critical challenges that remain to be addressed.

The first and foremost challenge deals with creating and effectively utilizing structure–function relationships on multiple length scales, as is readily found within naturally occurring hierarchical materials. Although research has not yet been able to achieve true hierarchical mimicry, combining several preparation techniques into one platform represents an interesting path forward to functional hierarchical nanocellulose‐based assemblies. In an example of utilizing alignment on multiple length scales, Magdassi et al. demonstrated a direct cryo‐writing (DCW) process to form aerogels, whereby CNC‐xyloglucan (XG) inks were printed onto a cold plate, facilitating directional freezing.^[^ ^213^ ^]^ Pore alignment was largely controlled by the rate of freezing, while the CNC:XG ratio governed mechanical properties and pore architecture, and the 3D‐printing process enabled the assembly of macroscale features. Although in this case, CNC alignment was largely uninvestigated, theoretically this could also add another level of functionality into such materials. In another recent example, Tripathi et al. demonstrated the preparation of aerogels from EISA CNC films, whereby the CNC chiral nematic order was preserved upon supercritical drying.^[^ ^261^ ^]^ Importantly, these anisotropic aerogels demonstrated greatly enhanced specific strength and specific toughness (properties which are generally mutually exclusive) versus isotropic aerogels.^[^ ^261^ ^]^ It then becomes clear that utilizing preparation techniques in combination with one another including wrinkling,^[^ ^262^ ^]^ solid/liquid templating,^[^ ^263^ ^]^ (pickering) emulsification,^[^ ^264^, ^265^, ^266^, ^267^, ^268^ ^]^ and lithographic processes^[^ ^269^ ^]^ could allow for the assembly of materials with structural order on multiple length scales, presenting the opportunity to create additional functionality which may traditionally be elusive. In addition, the integration of living organisms within bottom‐up fabrication processes also represents an interesting strategy for the formation of more precise/sophisticated architectures, which could lead to further improvements in material properties. For example, Schaffner et al. demonstrated a technique for 3D‐printing living bacteria into functional composites; here “living materials” with the ability to degrade pollutants or produce bacterial cellulose as a direct result of the embedded bacteria were successfully produced.^[^ ^270^ ^]^ Greca et al. similarly demonstrated the ability to successfully direct bacterial cellulose production in 3D over multiple length scales via a biofabrication technique acting to template and stabilize air–water interfaces, where production occurs.^[^ ^271^ ^]^ Therefore, co‐assembly techniques such as these, utilizing both sophisticated synthetic fabrication strategies and those present within living organisms represents an interesting opportunity to gain further control over the structure and functionality of assembled materials.

Finally, although nanocellulose has a large inherent potential as a building block for bottom‐up fabrication, it remains challenging to achieve optimal performance out of the particles on the materials scale, due to the necessity for near‐perfect organization across the nano and microscale (again, a feat in which nature excels and synthetic processes still lack). In contrast, there has been some recent work preparing nanocellulose‐based assemblies through top‐down fabrication processes, which benefits from utilizing the pre‐existing near‐perfect hierarchical arrangement of nanocellulose building blocks. Work done in the Burgert and Berglund labs have demonstrated the use of wood as a scaffold/template for functionalization via a delignification process, allowing for polymer impregnation while maintaining the hierarchical wood macrostructure;^[^ ^272^ ^]^ depending on the polymer added and method of addition, wood scaffolds can be readily prepared with optical transparency,^[^ ^273^, ^274^ ^]^ controllable mechanical gradients,^[^ ^275^ ^]^ stimuli responsiveness,^[^ ^276^ ^]^ or superhydrophobicity.^[^ ^277^ ^]^ The Hu lab has also demonstrated the preparation of delignified wood hydrogels, prepared via NaClO_2_ pretreatment and subsequent acrylamide infiltration and thermally initiated free radical polymerization.^[^ ^278^ ^]^ In this work, the existing alignment of CNF within the wood samples is effectively utilized in order to imbue anisotropy within the final wood‐based hydrogels, which display a tensile strength and elastic modulus of 36 and 310 MPa in the direction parallel to CNF alignment (representing some of the highest values ever demonstrated in any hydrogel material), and 0.54 and 0.135 MPa in the perpendicular direction.^[^ ^278^ ^]^ However, despite these advances in top‐down fabrication using wood as a scaffold, bottom‐up fabrication still possesses several advantages including easier dispersion of composite materials (within bulk and on surfaces) and the ability to introduce more homogenous functionality. Although bottom‐up fabrication is production‐intensive, and currently has difficulty bridging hierarchical structures across many length scales in the same material, these processes allow for more flexibility in material structuring, further allowing for the creation of biomimetic features but also completely novel structures, representing a promising path forward for the creation of truly next‐gen advanced materials.

## Conflict of Interest

The authors declare no conflict of interest.
